# Association of serum levels of antibodies against ALDOA and FH4 with transient ischemic attack and cerebral infarction

**DOI:** 10.1186/s12883-021-02301-w

**Published:** 2021-07-09

**Authors:** Hao Wang, Hao Lu, Xiao-Meng Zhang, Ken-ichiro Goto, Eiichi Kobayashi, Yoichi Yoshida, Akihiko Adachi, Tomoo Matsutani, Yasuo Iwadate, Seiichiro Mine, Toshio Machida, Mizuki Sata, Kazumasa Yamagishi, Hiroyasu Iso, Norie Sawada, Shoichiro Tsugane, Ikuo Kamitsukasa, Takeshi Wada, Akiyo Aotsuka, Kazuo Sugimoto, Hirotaka Takizawa, Koichi Kashiwado, Hideo Shin, Go Tomiyoshi, Rika Nakamura, Natsuko Shinmen, Hideyuki Kuroda, Anding Xu, Takaki Hiwasa

**Affiliations:** 1 Stroke Center, the First Affiliated Hospital, Jinan University, NO. 613, West Huangpu Ave., Tianhe Dist., Guangzhou, 510630 China; 2 Department of Biochemistry and Genetics, Graduate School of Medicine, Chiba University, Chiba, 260-8670 Japan; 3 Department of Neurological Surgery, Graduate School of Medicine, Chiba University, Inohana 1-8-1, Chuo-ku, Chiba, 260-8670 Japan; 4 Comprehensive Stroke Center, Chiba University Hospital, Chiba, 260-8677 Japan; 5 Department of Neurological Surgery, Chiba Prefectural Sawara Hospital, Chiba, 287-0003 Japan; 6 Department of Neurological Surgery, Chiba Cerebral and Cardiovascular Center, Chiba, 290-0512 Japan; 7 Department of Neurosurgery, Eastern Chiba Medical Center, Chiba, 283-8686 Japan; 8 Department of Public Health, Social Department of Social and Environmental Medicine, Graduate School of Medicine, Osaka University, Suita, Japan; 9 Department of Public Health Medicine, Faculty of Medicine, University of Tsukuba, Tsukuba, Japan; 10 Epidemiology and Prevention Group, Center for Public Health Sciences, National Cancer Center, Tokyo, Japan; 11 Department of Neurology, Chiba Rosai Hospital, Chiba, 290-0003 Japan; 12 Department of Neurology, Chibaken Saiseikai Narashino Hospital, Chiba, 275-8580 Japan; 13 Department of Internal Medicine, Chiba Aoba Municipal Hospital, Chiba, 260-0852 Japan; 14 Department of Neurology, Dongzhimen Affiliated Hospital, Beijing University of Chinese Medicine, Beijing, 100700 China; 15 Port Square Kashiwado Clinic, Kashiwado Memorial Foundation, Chiba, 260-0025 Japan; 16 Department of Neurology, Kashiwado Hospital, Chiba, 260-0854 Japan; 17 Department of Neurosurgery, Higashi Funabashi Hospital, Chiba, 274-0065 Japan; 18 Medical Project Division, Research Development Center, Fujikura Kasei Co., Saitama, 340-0203 Japan

**Keywords:** Transient ischemic attack, Cerebral infarction, ALDOA, FH, Antibody biomarker

## Abstract

**Background:**

Ischemic stroke, including transient ischemic attack (TIA) and acute-phase cerebral infarction (aCI), is a serious health problem in the aging society. Thus, this study aimed to identify TIA and aCI biomarkers.

**Methods:**

In 19 patients with TIA, candidate antigens recognized by serum IgG autoantibodies were screened using a human aortic endothelial cell cDNA library. Through amplified luminescent proximity homogeneous assay-linked immunosorbent assay (AlphaLISA), serum antibody levels against the candidate antigens were examined in healthy donor (HD), TIA, and aCI cohorts (*n* = 285, 92, and 529). The plasma antibody levels in the Japan Public Health Center-based Prospective Cohort Study (1991–1993) were also examined.

**Results:**

The candidate antigens were aldolase A (ALDOA) and fumarate hydratase (FH). In AlphaLISA, patients with TIA or aCI had higher anti-ALDOA antibody (ALDOA-Ab) and anti-FH antibody (FH-Ab) levels than the HDs (*P* < 0.05). In a multivariate logistic regression analysis, the ALDOA-Ab (odds ratio [OR]: 2.46, *P* = 0.0050) and FH-Ab (OR: 2.49, *P* = 0.0037) levels were independent predictors of TIA. According to the case–control study, the ALDOA-Ab (OR: 2.50, *P* < 0.01) and FH-Ab (OR: 2.60, *P* < 0.01) levels were associated with aCI risk. In a correlation analysis, both ALDOA-Abs and FH-Abs were well associated with hypertension, coronary heart disease, and habitual smoking. These antibody levels also correlated well with maximum intima–media thickness, which reflects atherosclerotic stenosis.

**Conclusions:**

ALDOA-Abs and FH-Abs can be novel potential biomarkers for predicting atherosclerotic TIA and aCI.

## Background

Ischemic stroke, including transient ischemic attack (TIA) and cerebral infarction (CI), is the most globally known cerebrovascular disorder. In particular, TIA is a temporary episode of neurological dysfunction caused by focal brain, spinal cord, or retinal ischemia, without acute infarction [1]. Meanwhile, CI is an episode of neurological dysfunction caused by focal brain infarction, often resulting in fatality and disability [2]. Patients with TIA are at a high risk of CI. According to epidemiologic studies, the prevalence of prior TIA in patients with CI was 15–30%. Additionally, the risk of CI on the 7th, 30th, and 90th-day post-TIA was 2.0 to 8.0%, 8.0 to 13.5%, and 9.5 to 20.1%, respectively [3, 4]. TIA with progressive aggravation is an early-warning signal for CI. Therefore, early TIA diagnosis and CI onset prediction are the key steps to reduce ischemic stroke occurrence [5].

Presently, TIA and CI can be predicted early in the medical field through several ways, including modern imaging techniques (e.g., transcranial Doppler [6], computed tomography, magnetic resonance imaging [7], and cerebral angiography [8]), blood biochemical indicators (e.g., oxidatively modified low-density lipoprotein [9], homocysteine [10], lipoprotein-related phospholipase A2, C-reactive protein [11], and heat shock protein [12]), and comprehensive assessment of risk factors [13] (e.g., hypertension, hyperlipidemia, body mass index [BMI], obesity, smoking habits, and family history). However, these methods are frequently insufficient to represent standard approaches for early TIA diagnosis and CI onset prediction. Therefore, novel biomarkers that would largely improve the management and prognosis of TIA and CI must be identified [14].

Atherosclerosis is highly likely to be involved in the pathogenesis of ischemic stroke, and most incident ischemic strokes (i.e., TIA and CI) are based on atherosclerosis [15]. Atherosclerosis is not only a simple pathological process of lipid deposition in the vascular wall. It is also an inflammatory proliferative dynamic mechanism induced by an excessive autoimmune response following vascular endothelial and smooth muscle cell injuries [16]. Endogenous antigens cause autoimmune responses that significantly influence the development of atherosclerosis, ultimately leading to the stenosis or blockage of the offending artery [17]. These antigens induce autoantibodies that have been detected in the serum of patients with atherosclerosis-related diseases, such as CI, coronary heart disease (CHD), and diabetes mellitus (DM) [18].

The serological identification of antigens by recombinant cDNA expression cloning (SEREX) is an established method for identifying endogenous antigenic proteins, combining molecular cloning and serological typing by using phage expression libraries [19]. SEREX was originally developed to screen out tumor-associated antigens, and it has identified more than 2300 novel tumor antigens recorded in Cancer Immunome Database, a public access online database [20, 21]. Hence, it is one of the most effective methods for identifying antigenic targets on a genome scale [22–30]. Consequently, it has also been used for autoimmune diseases, such as systemic lupus erythematosus, Kawasaki disease, Behcet’s disease, and multiple sclerosis [22–25]. In earlier studies, we used SEREX for examining atherosclerosis-related diseases and for identifying antibodies against RPA2 [26], MMP1, CBX1, and CBX5 [27] in CI, and ATP2B4, BMP-1 [28], TUBB2C [29], and SH3BP5 [30] in other atherosclerosis-related diseases.

Both TIA and CI have the pathological basis of atherosclerosis [15], and through SEREX, we found that atherosclerosis causes the increase of serum autoantibody levels in the early stage of lesions [26–30]. Clearly, the identification of sensitive, specific, and novel biomarkers is crucial to early predict TIA and CI. Therefore, this study aimed to identify autoantibodies associated with TIA and CI via SEREX. These autoantibodies could be used as molecular predictive biomarkers to reflect disease status.

## Methods

### Serum of patients and healthy donors (HDs)

We collected serum samples from HDs and patients diagnosed with TIA or CI caused by the development of atherosclerotic vulnerable plaque [31, 32]. HDs were selected from individuals with no history of TIA or CI, including acute-phase cerebral infarction (aCI) or old (chronic-phase) cerebral infarction (oCI), and with medical checkups, including cerebral MRI. Conversely, subjects with autoimmune disease were excluded. Next, we randomly selected 19 TIA serum samples, which were previously used to search for other stroke markers [27], and used them for SEREX screening.

In comparing the serum antibody levels, we set up four independent groups, which included 621 patients and 285 HDs. Of the 621 patients, 92, 464, and 65 suffered from TIA, aCI, and oCI, respectively. Table 1 shows the baseline characteristics of participants.

**Table 1:** Baseline characteristics of subjects enrolled in the study

	SEREX	AlphaLISA			
	TIA^a^ (*n* = 19)	Stroke			HD (*n* = 285)
		TIA (*n* = 92)	aCI (*n* = 464)	oCI (*n* = 65)	
Age	68.3*** (± 10.2)	70.2*** (± 11.6)	75.5*** (± 11.5)	73.3*** (± 9.2)	52.3 (± 11.7)
Male sex	16 (84.2%)	55 (59.7%)	271 (58.4%)	48 (73.8%)	188 (65.9%)
HT	13*** (68.4%)	60*** (65.2%)	335*** (72.2%)	53*** (81.5%)	57 (20.0%)
DM	3*** (15.8%)	27*** (29.3%)	125*** (26.9%)	22*** (33.8%)	11 (3.9%)
HL	3 (15.8%)	36*** (39.1%)	122*** (26.3%)	25*** (38.5%)	40 (14.0%)
CHD	1*** (5.2%)	5*** (5.4%)	40*** (8.6%)	2*** (3.1%)	0
Obesity (BMI ≥ 25)	10 (52.6%)	30 (32.6%)	127 (27.4%)	11 (16.9%)	88 (30.9%)
Smoking	12 (63.1%)	43 (46.7%)	228 (49.1%)	33 (50.8%)	132 (46.3%)

Data represents means (± SD) for continuous data and n (%) for categorical data

^a^*TIA* transient ischemic attack, *aCI* acute cerebral infarction, *HD* healthy donor, *oCI* old cerebral infarction, *HT* hypertension, *DM* diabetes mellitus, *HL* hyperlipidemia, *CHD* coronary heart disease, *BMI* body mass index

^***^ *P* < 0.001 *vs.* HD

Sera were extracted from the patients with TIA, aCI, and oCI in Chiba Prefectural Sawara Hospital, Chiba Rosai Hospital, and Chiba Aoba Municipal Hospital and from HDs in Chiba Prefectural Sawara Hospital, Higashi Funabashi Hospital, and Port Square Kashiwado Clinic. We centrifuged the samples at 3000 *g* for 10 min at room temperature and stored the supernatants at −80°C until use. Repeated thawing and freezing of samples were avoided.

### Clinical data

Regarding the risk factors of atherosclerosis, we collected the following data from the patients’ clinical records: age, sex, HT, DM, hyperlipidemia, CHD, obesity, and smoking. In this study, hypertension was defined as a history of systolic blood pressure > 140 mmHg, diastolic blood pressure > 90 mmHg, or the use of antihypertensive agents. DM was defined as having previously diagnosed with DM, treated with DM medication, and/or a fasting blood glucose level ≥ 126 mg/dL. Hyperlipidemia was defined as a history of total cholesterol > 220 mg/dL, triglyceride > 150 mg/dL, or the use of lipid-lowering agents. CHD was defined as a history of myocardial infarction or angina pectoris. Patients were considered as smokers if they either smoked during the study period or had a history of smoking. Finally, obesity was defined as BMI ≥ 25 kg/m^2^. We also collected the participants’ serum routine examination results, including blood routine, serum biochemistry, and blood electrolytes.

### Screening by expression cloning and identified antigens of sequence analysis

Clones that were immunoreactive against the serum of patients with TIA were screened using a commercially available human aortic endothelial cell cDNA library (Uni-ZAP XR Premade Library, Stratagene, La Jolla, CA). *Escherichia coli* (*E. coli*) XL1-Blue MRF′ was infected with Uni-ZAP XR phage. Further details were described in our previously published and improved version of the immunoscreening method [18, 26, 30, 33, 34].

The monoclonalized phage cDNA clones were converted into pBluescript phagemids by in vivo excision using ExAssist helper phage (Stratagene). Plasmid DNA was obtained from the *E. coli* SOLR strains transformed by the phagemids. We sequenced the inserted cDNAs, followed by homologous analysis using a public database provided by the National Center for Biotechnology Information (https://blast.ncbi.nlm.nih.gov/Blast.cgi).

### Purification of recombinant candidate proteins

To construct the expression plasmids of glutathione-S-transferase (GST)-fused proteins, we recombined the cDNA sequences into pGEX-4 T vectors and transfected them into *E. coli* BL-21, as previously described [6, 17, 21, 33, 34].

Subsequently, we cultured transformed *E. coli* BL-21 cells containing pGEX-4 T-2 clones and centrifuged the cell lysates. The GST-fusion recombinant proteins recovered in the supernatant fraction were directly purified by glutathione-Sepharose affinity chromatography (GE Healthcare Life Sciences) according to the manufacturer’s and our previous instructions [26, 28, 30]. We dissolved the precipitates containing recombinant proteins in 8 M urea in TED buffer [50 mM Tris–HCl (pH 8.0), 1 mM EDTA, and 1 mM dithiothreitol]. Next, we dialyzed the samples stepwise against 4 and 2 M urea in TED buffer every hour and then against the TED buffer. Finally, the recombinant proteins recovered in the supernatant using glutathione-Sepharose were purified, as described above [26–30].

### Western blotting

GST, GST–aldolase A (ALDOA), and GST–fumarate hydratase (FH) proteins (0.3 μg) were electrophoresed through SDS–polyacrylamide gel and analyzed by western blotting. To this end, we used anti-GST (goat) or 1:5000-diluted serum from patients with TIA or CI (#350 and #692). The proteins were then incubated with horseradish peroxidase-conjugated secondary antibody, as previously described [30, 33, 35, 36].

### Amplified luminescent proximity homogeneous assay-linked immunosorbent assay (AlphaLISA) of antibody biomarkers

The serum antibodies against the purified proteins were quantitatively measured by AlphaLISA. After being prepared according to Perkin Elmer’s instructions (Waltham, MA) and our previous reports [27, 30, 33, 34], the samples mixture was incubated for 14 days at room temperature in the dark. The chemical emission was read using the EnSpire Alpha microplate reader (Perkin Elmer). Specific reactions were calculated by subtracting the alpha values (alpha-photon counts) of the GST control from those of GST-fusion proteins.

### Nested case–control study

A nested case–control study was conducted using the antibody levels detected by AlphaLISA. This study was nested within the Japan Public Health Center (JPHC)-based Prospective Study [37, 38], which stored the plasma samples of approximately 30,000 Japanese individuals aged 40–69 years at a baseline period of 1990–1994. We used the samples of 202 incidental cases of acute ischemic stroke developed between the baseline and 2008 and those of 202 controls. The age (within 2 years), sex, blood sampling date (within 3 months), time since last meal (within 4 h), and study location (Public Health Center area) of these controls were matched with those of the cases. The odds ratios (ORs) and 95% confidence intervals (CIs) were estimated using a conditional logistic regression model. We informed the study participants of the objectives and methods of the study; those who answered the questionnaire and donated blood indicated that they gave informed consent to participate. The ethics committee of the National Cancer Center, Osaka University, and Tsukuba University approved this study.

### Statistical analyses

We compared differences in the alpha values between two groups by using Student’s *t*-test and Mann–Whitney *U* test. Additionally, the correlation between the alpha values and clinical case data was determined by Spearman’s correlation analysis. Using univariate and multivariate logistic regression analyses, we identified the set of variables that could be used to classify participants according to positive history for ischemic stroke. Furthermore, the ORs of the antibody levels of ALDOA and FH (ALDOA-Ab and FH-Ab, respectively) for CI in the nested case–control study were estimated and compared using a conditional logistic regression model. Through a receiver operating characteristic (ROC) analysis, we assessed the predictive values of markers for TIA and CI. Additionally, the cutoff values were set to maximize the sum of sensitivity and specificity. All tests were two-tailed. We considered *P* < 0.05 as statistically significant. All statistical data were analyzed using either the SPSS 13.0 software (SPSS Inc., Chicago, IL) or GraphPad Prism 5 (GraphPad Software, La Jolla, CA).

## Results

### Identification of ALDOA and FH as antigens in the serum of patients with TIA

**Fig. 1:**
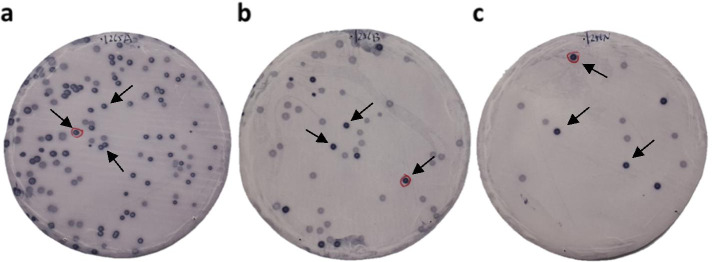
Immunoscreening of TIA antigens by SEREX. Bacterial proteins including phage cDNA products were blotted on nitrocellulose membranes and reacted with the sera of patients with TIA. The **a**, **b**, and **c** show the representative results of stained membranes (membrane diameter: 86 mm) after the second screening of SEREX. Arrows indicate positive phage clones

By expression cloning, two independent clones were identified in the serum of 19 patients with TIA (Fig. 1). Specifically, we found a sequence homology with ALDOA (Accession number: NM_184041) and FH (Accession number: NM_000143). The region between amino acids 70 and 469 of ALDOA and that between amino acids 1 and 185 of FH were obtained as pBluescript II clones; both were then recombined into pGEX-4 T-2 vectors individually. Recombinant ALDOA and FH proteins were expressed in *E. coli* as GST-fusion proteins and subsequently purified by glutathione-Sepharose affinity chromatography.

### Presence of serum antibodies confirmed by western blotting

**Fig. 2:**
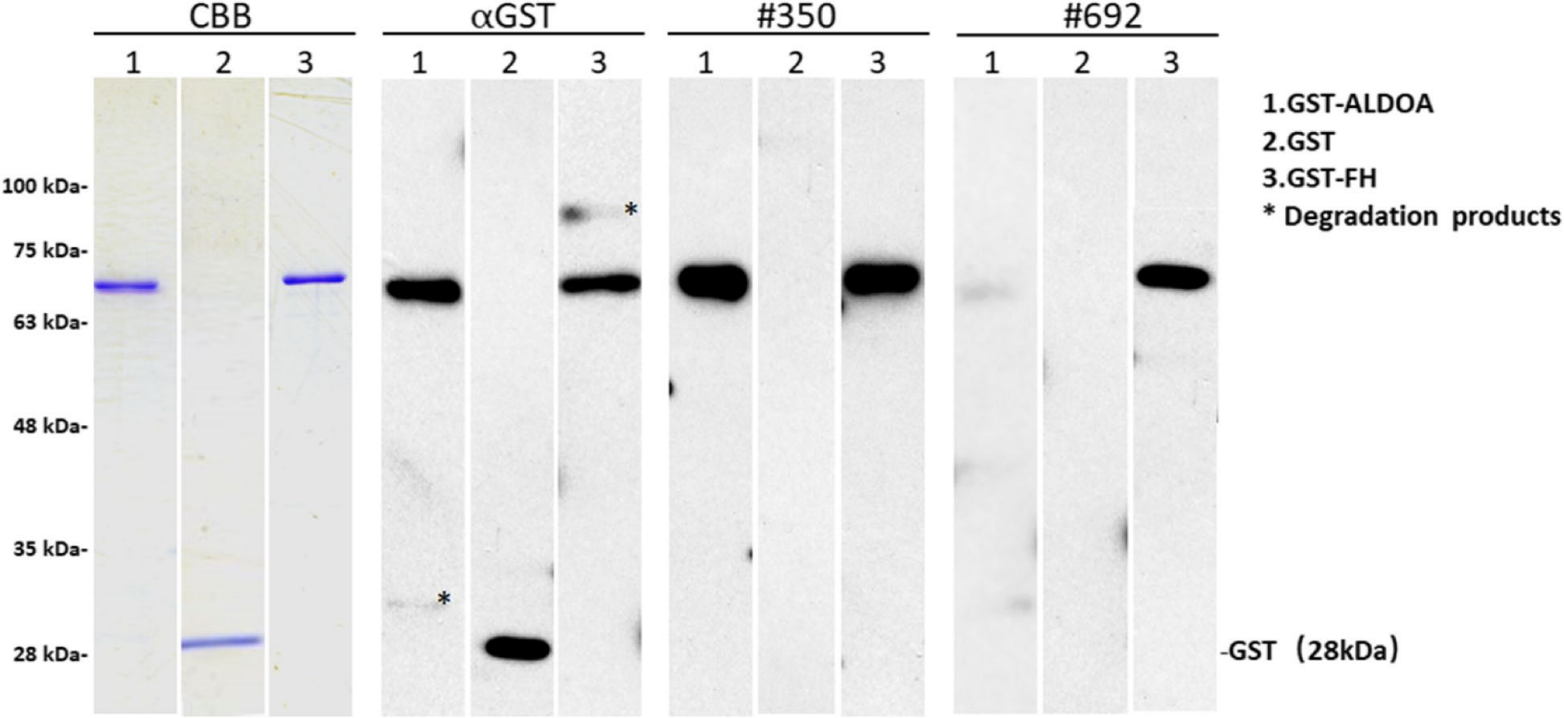
Presence of serum antibodies against ALDOA and FH antigenic proteins. The representative results of western blotting are shown. The latter showed the detection at the expected size of all the affinity-purified glutathione-S-transferase (GST)-fusion antigenic proteins (GST-ALDOA: 65 kDa; GST-FH: 67 kDa). GST and GST-fusion proteins were electrophoresed through SDS–polyacrylamide gels. They were subsequently stained with Coomassie Brilliant Blue or western blotting using anti-GST (αGST) or patient sera (#350 and #692). The specific reactions to GST-ALDOA and GST-FH are shown. The star represents degradation products after the electrophoresis. The left side of the figure shows the molecular weights

We aimed at confirming the presence of ALDOA-Abs and FH-Abs in the serum of patients with TIA or CI through western blotting. Using an anti-GST antibody, we recognized GST-ALDOA, GST-FH, and GST proteins as the reactions of 65-, 67-, and 28-kDa proteins, respectively (Fig. 2). Conversely, GST-ALDOA and/or GST-FH, but not GST, reacted with the serum antibodies of patients #350 and #692. Therefore, most, if not all, of the GST-fusion antigen proteins’ reactivity with serum antibodies may be caused by antigen proteins rather than the GST domain. Specific reactions against ALDOA or FH proteins were estimated by subtracting the antibody levels of GST from those of GST-tagged antigen proteins.

### Increase of ALDOA-Ab and FH-Ab levels in patients with TIA or CI

**Fig. 3:**
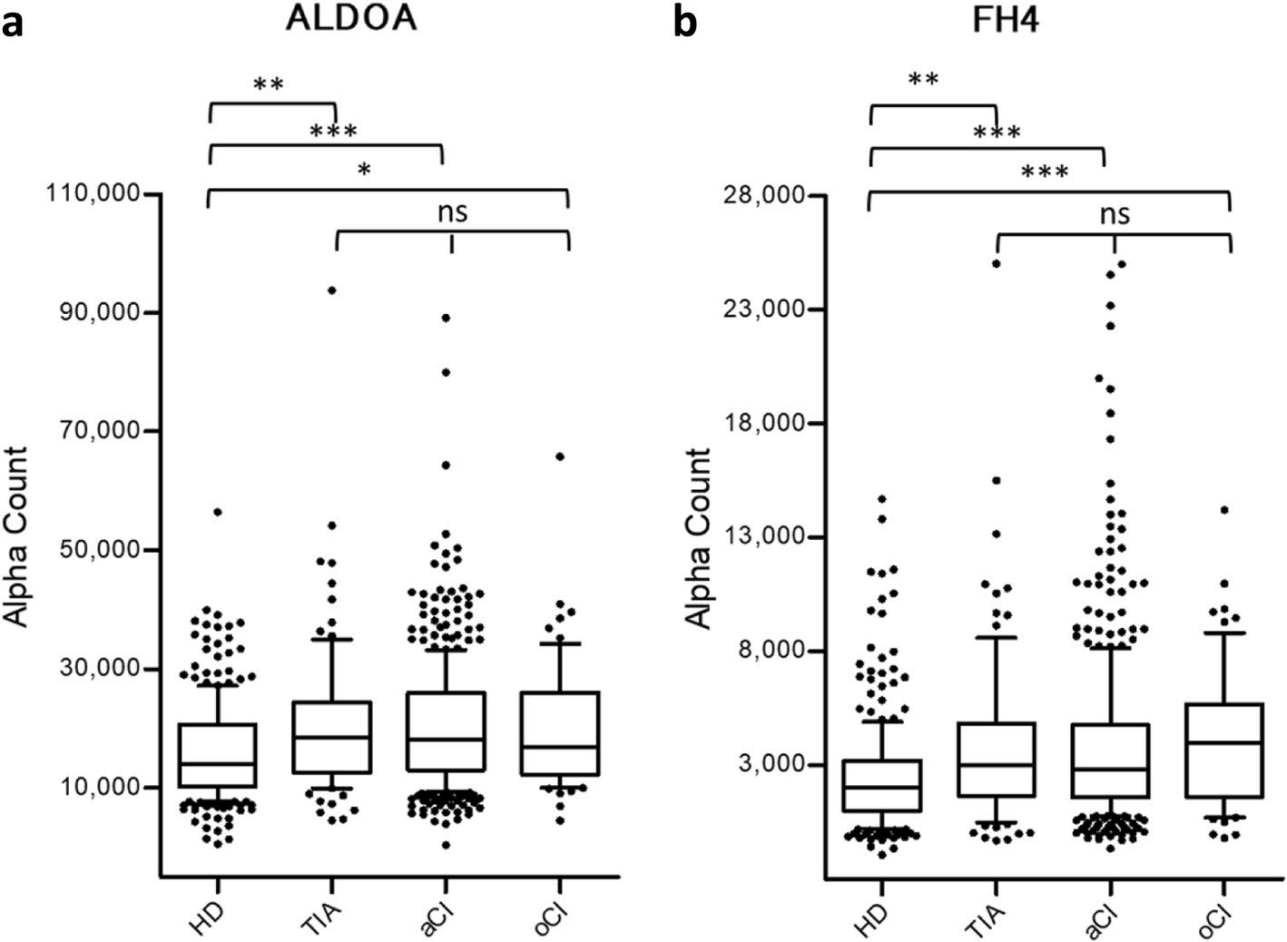
Comparison of the serum ALDOA-Ab and FH-Ab levels between HDs and patients with TIA, aCI, or oCI. The antigens GST-ALDOA (**a**) and GST-FH (**b**) were used. After the subtraction of the levels of antibodies against control GST, the serum antibody levels were examined by AlphaLISA, shown using a box-whisker. The stars indicate *P* values vs. HD specimens. One star indicates *P* < 0.05, two stars indicate *P* < 0.01, and three stars indicate *P* < 0.001. Table 2 shows the averages, SDs, total numbers, and *P* values

As mentioned, the ALDOA-Ab and FH-Ab levels in the sera of HDs and patients with TIA, aCI, or oCI were quantitatively analyzed using AlphaLISA. The alpha counts represent the luminescent photon counts corresponding to the antibody levels. Both antibody levels were significantly higher in patients with TIA, aCI, and oCI than in HDs (*P* < 0.05) (Table 2). Furthermore, the alpha counts were not significantly different between the three patient groups (Fig. 3). Therefore, ALDOA-Ab and FH-Ab levels may be closely related to the three ischemic cerebrovascular diseases (i.e., TIA, aCI, and oCI), but not to the disease type.

**Table 2:** Comparison of serum antibody levels between HDs and patients with TIA, aCI, or oCI examined by AlphaLISA

	HD		TIA		aCI		oCI	
	ALDOA-Ab	FH-Ab	ALDOA-Ab	FH-Ab	ALDOA-Ab	FH-Ab	ALDOA-Ab	FH-Ab
Average	16,326	2,492	20,675	3,852	20,431	3,850	20,144	4,226
SD	10,410	2,367	12,464	3,765	10,564	3,699	10,633	3,112
Total number	285	285	92	92	464	464	65	65
*P* (vs. HD)			**0.0030**	**0.0015**	** < 0.0001**	** < 0.0001**	**0.0102**	** < 0.0001**

The average, SD, and the total sample number are presented for HDs and patients as well as *P* values of statistical comparisons between HDs and patients. *P* values less than 0.05 are marked in bold

**Fig. 4:**
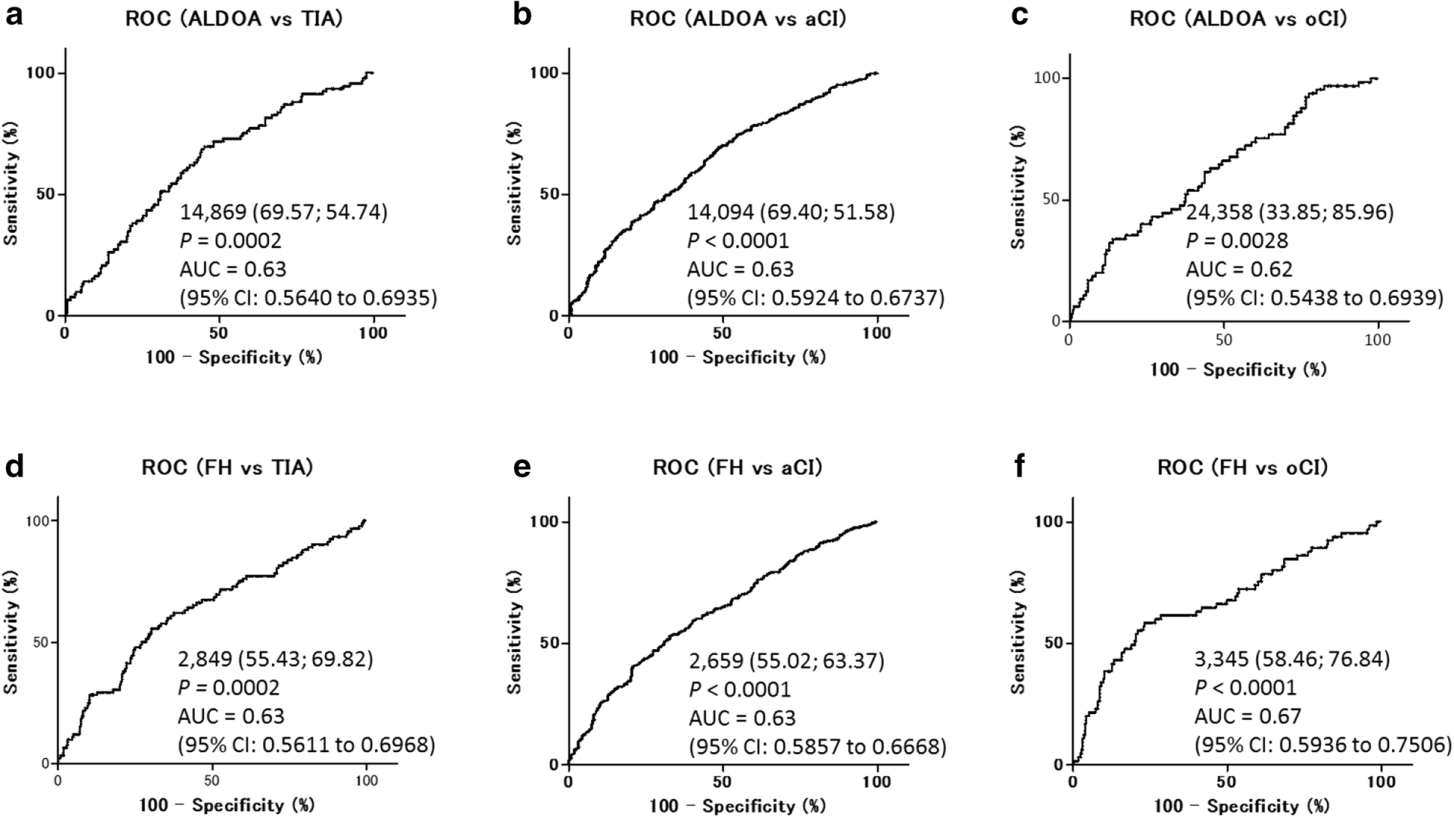
Receiver operating characteristic (ROC) analysis of ALDOA-Abs and FH-Abs as predictors of TIA, aCI, or oCI. Numbers in the figures indicate the cutoff values for marker levels. Numbers in parentheses indicate sensitivity (left) and specificity (right). Areas under the curve (AUC), 95% confidence intervals (95% CI), and *P* values are shown

The ability of the markers ALDOA-Abs and FH-Abs to detect TIA, aCI, and oCI was evaluated by the ROC analysis. The areas under the curve of ALDOA-Abs and FH-Abs were 0.63 (95% CIs: 0.56–0.69) (Fig. 4a) and 0.63 (95% CI: 0.56–0.70) (Fig. 4d) for TIA, 0.63 (95% CI: 0.60–0.67) (Fig. 4b) and 0.63 (95% CI: 0.59–0.67) (Fig. 4e) for aCI, and 0.62 (95% CI: 0.54–0.70) (Fig. 4c) and 0.67 (95% CI: 0.60–0.75) (Fig. 4f) for oCI, respectively. At a cutoff value of 14,869 for the ALDOA-Abs, the antibody level’s sensitivity and specificity for TIA diagnosis were 69.57 and 54.74%, respectively (Fig. 4a), similar to those for aCI diagnosis (69.40 and 51.58%, respectively) (Fig. 4b). The sensitivity and specificity for FH-Abs are shown in Fig. 4d, e, and f.

### Association between TIA and clinical parameters including ALDOA-Ab and FH-Ab levels

Table 3 summarizes the results of univariate and multivariate logistic regression analyses. Using the cutoff values of 14,869 and 2849, the univariate logistic regression analysis revealed that the elevated ALDOA-Ab (OR: 2.91, 95% CI: 1.76–4.83, *P* < 0.0001) and FH-Ab (OR: 2.88, 95% CI: 1.78–4.67, *P* < 0.0001) levels were associated with the increased risk for TIA, respectively. Those factors with *P* < 0.05 in the univariate analysis were used in the multivariate regression analysis. In this subsequent analysis, elevated ALDOA-Ab (OR: 2.46, 95% CI: 1.31–4.62, *P* = 0.0050) and FH-Ab (OR: 2.49, 95% CI: 1.35–4.63, *P* = 0.0037) levels were independent predictors of TIA. The predictive values of ALDOA-Abs and FH-Abs for TIA were similar to that of hypertension, which is a typical risk factor of TIA. Specifically, the risk factors of TIA were as follows: age (OR: 6.04, 95% CI: 3.15–11.58, *P* < 0.0001), hypertension (OR: 2.97, 95% CI: 1.61–5.45, *P* = 0.0005), and DM (OR: 5.31, 95% CI: 2.05–13.79, *P* = 0.0006).

**Table 3:** Logistic regression of predictive factors for TIA (*n* = 377; no. of events = 92)

	Univariate			Multivariate		
	*P*	OR	95% CI^a^	*P*	OR	95% CI
Age (≥ 60)	< 0.0001	**9.97**	5.65–17.59	< 0.0001	**6.04**	3.15—11.58
Sex	0.2304	1.34	0.83–2.17			
HT	< 0.0001	**7.50**	4.47–2.59	0.0005	**2.97**	1.61—5.45
DM	< 0.0001	**10.35**	4.88–21.94	0.0006	**5.31**	2.05—13.79
HL	< 0.0001	**3.94**	2.30–6.73	0.0523	1.94	0.99—3.79
CHD	0.0132	**8.13**	1.55–42.66	0.8917	1.14	0.17—7.77
BMI (≥ 25)	0.7768	1.08	0.65–1.80			
Smoking	0.9653	1.01	0.63–1.62			
ALDOA-Ab^a^	< 0.0001	**2.91**	1.76–4.83	0.0050	**2.46**	1.31—4.62
FH-Ab^b^	< 0.0001	**2.88**	1.78–4.67	0.0037	**2.49**	1.35—4.63

Cutoff values of ADOLA-Abs and FH-Abs were 14,869 and 2,849, respectively, based on ROC curve analysis

OR values > 2.00 are marked in bold

^a^*95% CI* 95% confidence interval, *OR* odds ratio

### Elevated positive predictive values (PPVs) by the combination of ALDOA-Abs, FH-Abs, and clinical risk factors

Next, we calculated the positive rates of 92 patients with TIA and 285 HDs, with the involvement of the conventional risk factors (i.e., age, HT, and DM). We used the cutoff values of 14,869 and 2849 for ALDOA-Abs and FH-Abs to detect TIA. The PPVs of age, HT, and DM were 48.0, 51.3, and 71.1%, respectively (Table 4). Conversely, the PPVs of ALDOA-Abs combined with age, HT, and DM increased to 63.1, 63.5, and 91.3%, whereas those of FH-Abs were 61.9, 56.9, and 94.1%, respectively, thereby similar. Furthermore, the PPVs of ALDOA-Abs combined with HT and DM, and those of FH-Abs combined with age and DM reached up to 100%.

**Table 4:** Validation of predictive factors for TIA (*n* = 377; number of events = 92)

		Age^c^	HT	DM	Age + HT	Age + DM	HT + DM	Age + HT + DM
Single risk	TIA ( +)	73	60	27	53	25	21	21
	TIA (-)	79	57	11	28	5	5	2
	PPV^d^	48.0%	51.3%	71.1%	65.4%	83.3%	80.8%	91.3%
Single risk + ALDOA-Ab^a^	TIA ( +)	53	47	21	41	20	17	17
	TIA (-)	31	27	2	12	1	0	0
	PPV	63.1%	63.5%	91.3%	77.4%	95.2%	100.0%	100.0%
Single risk + FH-Ab^b^	TIA ( +)	39	33	16	29	14	14	13
	TIA (-)	24	25	1	12	0	1	0
	PPV	61.9%	56.9%	94.1%	70.7%	100.0%	93.3%	100.0%
Single risk + ALDOA-Ab + FH-Ab	TIA ( +)	32	29	15	25	14	13	13
	TIA (-)	11	14	0	7	0	0	0
	PPV	74.4%	67.4%	100.0%	78.1%	100.0%	100.0%	100.0%

^a^ADOLA-Ab, elevated ADOLA-Ab levels, > 14,869

^b^FH-Ab, elevated FH-Ab levels, > 2,849

^c^Age, ≥ 60

^d^PPV, positive predictive value

### JPHC cohort analysis

The abovementioned logistic regression analysis proved that ALDOA-Abs and FH-Abs are independent early-warning risk factors of TIA, which is one of prodromal stages of CI. To further validate their association with CI, we conducted a prospective case–control study nested within the JPHC-based Prospective Study (the interference of age, sex, and area was excluded). The ALDOA-Ab and FH-Ab levels were divided into quartiles. For the participants with the second and highest quartiles of the antibody level, the ORs (95% CIs) were 2.38 (1.24–4.55) and 2.50 (1.26–4.96), respectively, compared with that for those with the lowest quartile (Table 5). Additionally, the FH-Ab levels were positively associated with the risk for aCI. Specifically, the ORs (95% CIs) were 2.17 (1.20–3.92) and 2.60 (1.41–4.80) for those participants with the third and highest quartiles of the antibody level, respectively. Therefore, ALDOA-Abs and FH-Abs can predict the onset of aCI.

**Table 5:** Age and sex-matched, conditional odds ratios and 95% confidence intervals of incident aCI according to antibody markers (202 cases and 202 controls)

Antibody marker		Case / Control	Matched OR (95% CI)
ALDOA-Abs	1st	30 / 50	1.00
	2nd	62 / 51	**2.38** (1.24—4.55)
	3rd	50 / 51	1.95 (1.00—3.82)
	4th	60 / 50	**2.50** (1.26—4.96)
FH-Abs	1st	29 / 50	1.00
	2nd	40 / 51	1.33 (0.72—2.48)
	3rd	62 / 51	**2.17** (1.20—3.92)
	4th	71 / 50	**2.60** (1.41—4.80)

OR values > 2.00 are marked in bold

### Association between the ALDOA-Ab and FH-Ab levels and the clinical parameters

We then examined whether the serum ALDOA-Ab and FH-Ab levels correlate with clinical parameters such as sex, other diseases, lifestyle, and obesity (Table 6). The ALDOA-Ab levels were well associated with blood pressure (*P* = 0.0022), CHD (*P* = 0.0400), and habitual smoking (*P* < 0.0001), but not with sex, DM, HL, alcohol intake, or obesity. The FH-Ab levels showed similar correlation with the ALDOA-Ab levels, except that the former significantly correlated with DM (*P* = 0.0183) and less correlated with smoking habit (*P* = 0.0566).

**Table 6:** Correlation analysis between serum antibody marker levels and sex, other diseases, lifestyle, and obesity

		ALDOA-Ab		FH-Ab	
Sex		Male	Female	Male	Female
Sample number		692	458	692	458
Antibody level	Average	19,087	18,196	3,323	3,492
	SD	11,046	10,456	3,258	3,630
*P* value (vs Male)			0.1941		0.2563
DM		DM (–)	DM ( +)	DM (–)	DM ( +)
Sample number		926	219	926	219
Antibody level	Average	18,637	19,189	3,315	3,710
	SD	11,045	9,902	3,425	3,349
*P* value [vs DM (–)]			0.1250		**0.0183**
Blood pressure		HT (–)	HT ( +)	HT (–)	HT ( +)
Sample number		505	640	505	640
Antibody level	Average	17,927	19,388	2,977	3,717
	SD	11,037	10,635	2,985	3,685
*P* value [vs HT (–)]			**0.0022**		** < 0.0001**
CHD		CHD (–)	CHD ( +)	CHD–	CHD +
Sample number		1086	59	1086	59
Antibody level	Average	18,600	21,373	3,336	4,397
	SD	10,685	13,087	3,351	4,302
*P* value [vs CHD (–)]			**0.0400**		**0.0268**
Lipidemia		HL (–)	HL ( +)	HL (–)	HL ( +)
Sample number		844	301	844	301
Antibody level	Average	18,871	18,384	3,514	3,045
	SD	11,144	9,923	3,688	2,455
*P* value [vs HL (–)]			0.6244		0.6545
Life style		Non-smoker	Smoker	Non-smoker	Smoker
Sample number		584	561	584	561
Antibody level	Average	17,371	20,158	3,282	3,494
	SD	9,641	11,783	3,587	3,186
*P* value (vs non-smoker)			** < 0.0001**		0.0566
Life style		Alcohol (–)	Alcohol ( +)	Alcohol (–)	Alcohol ( +)
Sample number		338	570	338	570
Antibody level	Average	17,928	19,507	3,528	3,466
	SD	9,311	12,373	3,544	3,492
*P* value [vs Alcohol (-)]			0.0817		0.8972
Obesity		BMI < 25	BMI ≥ 25	BMI < 25	BMI ≥ 25
Sample number		809	315	809	315
Antibody level	Average	18,911	18,568	3,577	2,992
	SD	11,198	10,049	3,722	2,475
*P* value (vs BMI < 25)			0.8354		0.0720

The subjects were divided into two groups as follows: sex (male and female); presence ( +) or absence ( −) of complication of DM, HT, CHD or hyperlipidemia (HL), lifestyle factors (smoking and alcohol intake habits), and obesity. Antibody levels (alpha counts) were compared using the Mann–Whitney *U* test. Sample numbers, averages and SDs of counts as well as *P* values are shown. Significant correlations (*P* < 0.05) are marked in bold

The correlation between the antibody levels and other clinical parameters was also examined by Spearman correlation analysis. The levels of both AODOA-Abs and FH-Abs significantly correlated with age, blood pressure, maximum intima–media thickness (max IMT), and blood sugar (Table 7). Considering that max IMT is one of indices of atherosclerosis, both ALDOA-Abs and FH-Abs could reflect the degree of atherosclerosis. Consistent with the results in Table 6, DM-related blood sugar was more closely related to FH-Abs than ALDOA-Abs. Meanwhile, the antibody levels inversely correlated with the albumin/globulin ratio, cholinesterase, total protein, albumin, total cholesterol, and red blood cell count.

**Table 7:** Correlation analysis between serum antibody marker levels and the indices in HDs and CI patients

	ALDOA		FH4	
	*r* value	*P* value	*r* value	*P* value
Age	0.1973	** < 0.0001**	0.2369	** < 0.0001**
Blood pressure	0.1574	** < 0.0001**	0.0919	**0.0196**
Max intima-media thickness	0.2353	** < 0.0001**	0.2179	** < 0.0001**
Albumin/globulin ratio	-0.1133	**0.0040**	-0.1107	**0.0049**
Aspartate transaminase	0.0282	0.4660	0.0236	0.5422
Alanine transaminase	-0.0366	0.3441	-0.0433	0.2631
Alkaline phosphatase	0.0609	0.1319	0.0699	0.0836
Lactate dehydrogenase	0.0634	0.1073	0.0495	0.2082
Total bilirubin	-0.0459	0.2419	-0.0369	0.3460
Cholinesterase	-0.1175	**0.0094**	-0.1991	** < 0.0001**
γ-Glutamyl transpeptidase	0.0558	0.1643	-0.0484	0.2274
Total protein	-0.1475	**0.0002**	-0.1048	**0.0076**
Albumin	-0.1652	** < 0.0001**	-0.1453	**0.0002**
Blood urea nitrogen	0.0340	0.3802	0.0538	0.1647
Creatinin	0.0058	0.8808	0.0146	0.7065
Glomerular filtration rate	-0.0044	0.9131	-0.0556	0.1715
Uric acid	0.0509	0.2716	-0.0233	0.6148
Amylase	-0.0890	0.0817	-0.0336	0.5113
Total cholesterol	-0.1453	**0.0005**	-0.1262	**0.0024**
High density lipoprotein cholesterol	-0.0658	0.1855	-0.0503	0.3125
Triglyceride	-0.0904	0.0579	-0.1335	**0.0050**
Na^+^	0.0684	0.0794	-0.0467	0.2316
K^+^	-0.0563	0.1495	-0.0217	0.5783
Cl^−^	0.0632	0.1052	0.0193	0.6220
C-reactive protein	0.1788	**0.0001**	0.0491	0.2915
White blood cell number	0.0909	**0.0188**	0.0737	0.0569
Red blood cell number	-0.0781	**0.0436**	-0.1152	**0.0029**
Hemoglobin	-0.0565	0.1447	-0.0984	**0.0110**
Hematocrit	-0.0506	0.1915	-0.0971	**0.0120**
Platelet number	-0.0335	0.3875	-0.0762	**0.0490**
Mean platelet volume	-0.0032	0.9334	-0.0021	0.9569
Plateletcrit	-0.0323	0.4047	-0.0902	**0.0197**
Platelet distribution width	-0.0174	0.6534	-0.0382	0.3245
Blood sugar	0.0861	**0.0327**	0.1512	**0.0002**
Glycated hemoglobin A1c	-0.0320	0.4758	-0.0183	0.6830

The data on study individuals were obtained from HD subjects in Chiba Prefectural Sawara Hospital, Higashi Funabashi Hospital, and Port Square Kashiwado Clinic and TIA, aCI or cCI patients in Chiba Prefectural Sawara Hospital, Chiba Rosai Hospital, and Chiba Aoba Municipal Hospital. Correlation coefficients (r) and *P* values were calculated via Spearman’s correlation analysis. In bold we marked *P* < 0.05

## Discussion

After the application of SEREX using the sera of patients with TIA, we found two antigens, namely, ALDOA and FH. Additionally, the presence of antibodies against ALDOA and FH in the patients’ sera was confirmed by western blotting (Fig. 2). We also evaluated the antibody levels through AlphaLISA, which allowed us to compare the levels between patients and HDs. Our results showed that compared with HDs, patients with TIA, aCI, or oCI had significantly elevated levels of ALDOA-Abs and FH-Abs (Fig. 3). We further found that these antibodies are independent predictors (the interference of age, sex, and area was excluded) of TIA by a clinical statistical analysis (Table 3). Of note, TIA tends to develop into aCI, thereby a clear risk factor of aCI [39]. As independent early-warning risk factors of TIA, the elevated ALDOA-Ab and FH-Ab levels may also be predictive markers of aCI. Therefore, we further confirmed it by the statistical analysis of prospective case–control studies nested in large community-based samples (Table 5).

ALDOA is one of the glycolytic enzymes that catalyze the reversible conversion of fructose-1,6-bisphosphate into glyceraldehyde-3-phosphate and dihydroxyacetone phosphate [40]. ALDOA is widely distributed in the entire body tissues. As a catalytic enzyme, ALDOA represents as one of the key enzymes in glycolysis. Of note, it participates in the hypoxic responses regulating both glucose and energy metabolisms and can be a hypoxia biomarker [41]. Ischemic stroke is a typical atherosclerosis-related disease, with local tissue hypoxia as its basic pathophysiological feature. ALDOA is also a hypoxia-inducible gene expression product [42]. When brain tissue undergoes ischemia or hypoxia, both glucose uptake and metabolism are stimulated to compensate for the reduced energy production by inducing ALDOA overexpression [43]. Hypoxia-inducible factor 1-alpha (HIF-1α), which is a transcription factor that is sensitive to hypoxia-inducible genes, upregulates ALDOA expression in hypoxic cells [44], thereby enhancing its glycolysis metabolism. Chang et al*.* proved that ALDOA overexpression upregulates the expression of matrix metalloproteinase (MMP) 9 via HIF-1α [45]. These results are in line with our previous study [27] wherein anti-MMP1 antibodies demonstrated increased specificity in the serum of patients with TIA. MMPs can degrade the main components of the vascular extracellular matrix and is an important factor responsible for the induction of atherosclerosis. A close relationship between ALDOA and MMPs suggests that ALDOA overexpression may not only be a sequential pathological process in TIA development; it may also be related to the initiation and deterioration of TIA. Moreover, the present study revealed the ALDOA-Ab levels were well associated with HT, CHD, and habitual smoking, but not with sex, DM, HL, alcohol abuse, and obesity (Table 6). ALDOA-Abs were also associated with max IMT, metabolic disturbance (e.g., blood sugar, total cholesterol, and total protein), and inflammation (e.g., white blood cell count and C-reactive protein) (Table 7). Thus, the serum ALDOA-Ab marker can discriminate HT-induced atherosclerotic TIA and aCI.

Meanwhile, FH is a key enzyme involved in the tricarboxylic acid (TCA) cycle. It can reversibly catalyze the conversion of fumaric acid into L-malate in the cells [46]. The TCA cycle mainly functions by oxidizing pyruvate supplied by the glycolytic pathway, with the goal of producing energy. Aside from its classical metabolic functions, FH has other nonmetabolic functions under cellular stimulation [47]. FH has been reported to be associated with tumorigenesis, specifically by altering the gene expression and configuration of tumor cells [48]. Xiao et al*.* found that FH could antagonize α-ketoglutarate-dependent demethylase through its metabolite fumarate, thereby affecting histone methylation [49]. In addition, Wang et al*.* showed that in the absence of glucose or hypoxia, which inhibits histone demethylation by lysine-specific demethylase 2A, FH exhibits adenosine monophosphate–activated protein kinase–mediated phosphorylation [50]. FH inhibits histone methylation by reducing the physiological activity of vascular endothelial growth factor (VEGF) [51]. This outcome, in turn, affects the repair and remodeling of the vascular endothelium after atherosclerosis. Of note, abnormal histone methylation is responsible for significant changes in the expression of genes, including *VEGF*. FH regulates the occurrence and development of atherosclerosis [52], consistent with our finding that the FH-Ab levels in patients with TIA and ischemic stroke are significantly higher than those in HDs (Fig. 3).

Hyperlipidemia contains the following three criteria: total cholesterol > 220 mg/dL, triglyceride > 150 mg/dL, or regular use of lipid-lowering agents; in addition, it is one of the risk factors for atherosclerosis [53]. However, the atherosclerosis markers ALDOA-Ab and FH-Ab were not associated with hyperlipidemia, but with hypertension (Tables 6 and 7). Atherosclerosis is a multifactorial disease [54], and the upstream atherosclerosis-inducing mechanism via hyperlipidemia could be different from that via hypertension, of which the latter pathway might be accompanied with the elevated expression of ALDOA and FH.

Given that TIA is one of the prodromal stages of aCI, ALDOA-Abs and FH-Abs could be used as risk predictors of TIA and aCI. To examine this hypothesis, we conducted a case–control study nested within the JPHC-based Prospective Study. The ALDOA-Ab and FH-Ab levels were measured in 202 cases of incident CI documented between the baseline and 2008, and in 202 controls with matching age, sex, and area. Subsequently, the ALDOA-Ab and FH-Ab levels for aCI (associated with atherosclerosis) were estimated using a conditional logistic regression model. Our results showed that both the antibody levels were positively and strongly associated with the risk for aCI (Table 5). Therefore, such antibody markers can be applied to predictive diagnosis rather than simple risk evaluation.

Although the PPVs of ALDOA-Abs and FH-Abs for TIA were 69.57% and 55.43%, respectively (Fig. 4), they increased when combined with conventional risk factors such as age, HT, and DM (Table 4). Especially, 100% of PPV was attained by combining ALDOA-Abs with HT and DM or combining FH-Abs with age and DM. Thus, antibody markers combined with clinical risk factors improve the ability to predict TIA, suggesting that this combination is also applicable to the early prediction of aCI. The ALDOA-Ab and FH-Ab levels correlated with CHD, HT, habitual smoking (ALDOA-Ab), DM (FH-DM) (Table 6), age, max IMT, C-reactive protein, and blood sugar (Table 7). Max IMT is one of typical indices of atherosclerosis, and CHD and DM are atherosclerosis-related diseases. Furthermore, HT, habitual smoking, age, C-reactive protein, and blood sugar are risk factors of atherosclerosis. Therefore, ALDOA-Abs and FH-Abs are biomarkers for atherosclerotic ischemic stroke. The large-scale JPHC cohort analysis also showed that these antibody markers were useful predictors of aCI onset.

## Conclusion

The levels of antibodies against ALDOA and FH were significantly higher in patients with TIA or aCI than in HDs. These antibody markers can be novel predictors of TIA and pre-onset aCI, which are induced by atherosclerosis.

## Data Availability

The datasets used and/or analyzed during the current study are available from the corresponding author on reasonable request.

## Abbreviations

TIA: Transient ischemic attack
aCI: Acute cerebral infarction
HD: Healthy donor
oCI: Old cerebral infarction
HT: Hypertension
DM: Diabetes mellitus
CHD: Coronary heart disease
BMI: Body mass index
SEREX: Serological identification of antigens by recombinant cDNA expression cloning
*E. coli*: *Escherichia coli*
GST: Glutathione-S-transferase
AlphaLISA: Amplified luminescent proximity homogeneous assay-linked immunosorbent assay
JPHC: The Japan Public Health Center-based Prospective Study
OR: Odds ratio
ROC: Receiver operating characteristic
ALDOA: Aldolase A, fructose-bisphosphate
ALDOA-Ab: Anti-ALDOA antibody
FH: Fumarate hydratase
FH-Ab: Anti-FH antibody
95% CI: 95% Confidence interval
PPV: Positive predictive value
max IMT: Maximum intima-media thickness
HIF-1α: Hypoxia-inducible factor 1α
MMP: Matrix metalloproteinase
VEGF: Vascular endothelial growth factor
